# Central venous oxygenation: when physiology explains apparent discrepancies

**DOI:** 10.1186/s13054-014-0579-9

**Published:** 2014-11-10

**Authors:** Pierre Squara

**Affiliations:** Clinique Ambroise Paré, 27 boulevard Victor Hugo, 92200 Neuilly-sur-Seine, France

## Abstract

Central venous oxygen saturation (ScvO_2_) >70% or mixed venous oxygen saturation (SvO_2_) >65% is recommended for both septic and non-septic patients. Although it is the task of experts to suggest clear and simple guidelines, there is a risk of reducing critical care to these simple recommendations. This article reviews the basic physiological and pathological features as well as the metrological issues that provide clear evidence that SvO_2_ and ScvO_2_ are adaptative variables with large inter-patient variability. This variability is exemplified in a modeled population of 1,000 standard ICU patients and in a real population of 100 patients including 15,860 measurements. In these populations, it can be seen how optimizing one to three of the four S(c)vO_2_ components homogenized the patients and yields a clear dependency with the fourth one. This explains the discordant results observed in large studies where cardiac output was increased up to predetermined S(c)vO_2_ thresholds following arterial oxygen hemoglobin saturation, total body oxygen consumption needs and hemoglobin optimization. Although a systematic S(c)vO_2_ goal-oriented protocol can be statistically profitable before ICU admission, appropriate intensive care mandates determination of the best compromise between S(c)vO_2_ and its four components, taking into account the specific constraints of each individual patient.

## Introduction

A recent review of the literature concluded that ‘central venous oxygen saturation (ScvO_2_) is a very important measurement, which can be easily taken in a critical care environment by both medical and nursing staff. It provides an understanding of the patient's oxygen delivery, oxygen consumption and cardiac output. It has a key role within early goal-directed therapy and has been shown to decrease mortality when taken and analyzed appropriately’ [1]. Indeed, ScvO_2_ > 70% or mixed venous oxygen saturation (SvO_2_) >65% is recommended for both septic and non-septic patients [2-4].

There is no debate that a major task of experts is to determine clear and simple rules for the early treatment of life-threatening disorders. As a consequence of the worldwide promotion of these recommendations, however, there is a risk of reducing intensive care to these simple protocols. The objective of this review is to highlight the basic physiological and pathological features as well as the metrological issues that provide clear evidence that, in reality, and for each specific patient, SvO_2_ and ScvO_2_ are complex dynamic variables that may not always provide an appropriate cutoff for all clinical settings [5-7]. This may explain that targeting unique S(c)vO_2_ thresholds may balance positive and negative effects and may produce hazardous results in large studies.

## The fundamental equilibrium

The *sine qua non* condition for adequate energy supply is that the circulatory system transports to each cell enough oxygen (O_2_), nutrients, and chemicals to ensure their aerobic respiration [8,9]. ‘Adequate’ means that, at any time, the difference (gap) between the expected metabolic needs minus the real O_2_ consumption (VO_2_) must not exceed energy storage. This basic equilibrium can be conveniently formulated as:

1 $$ \mathrm{V}{\mathrm{O}}_2\left(\mathrm{measured}\right) = \mathrm{V}{\mathrm{O}}_2\mathrm{needs} - \mathrm{V}{\mathrm{O}}_2\mathrm{gap} $$

**Figure 1:**
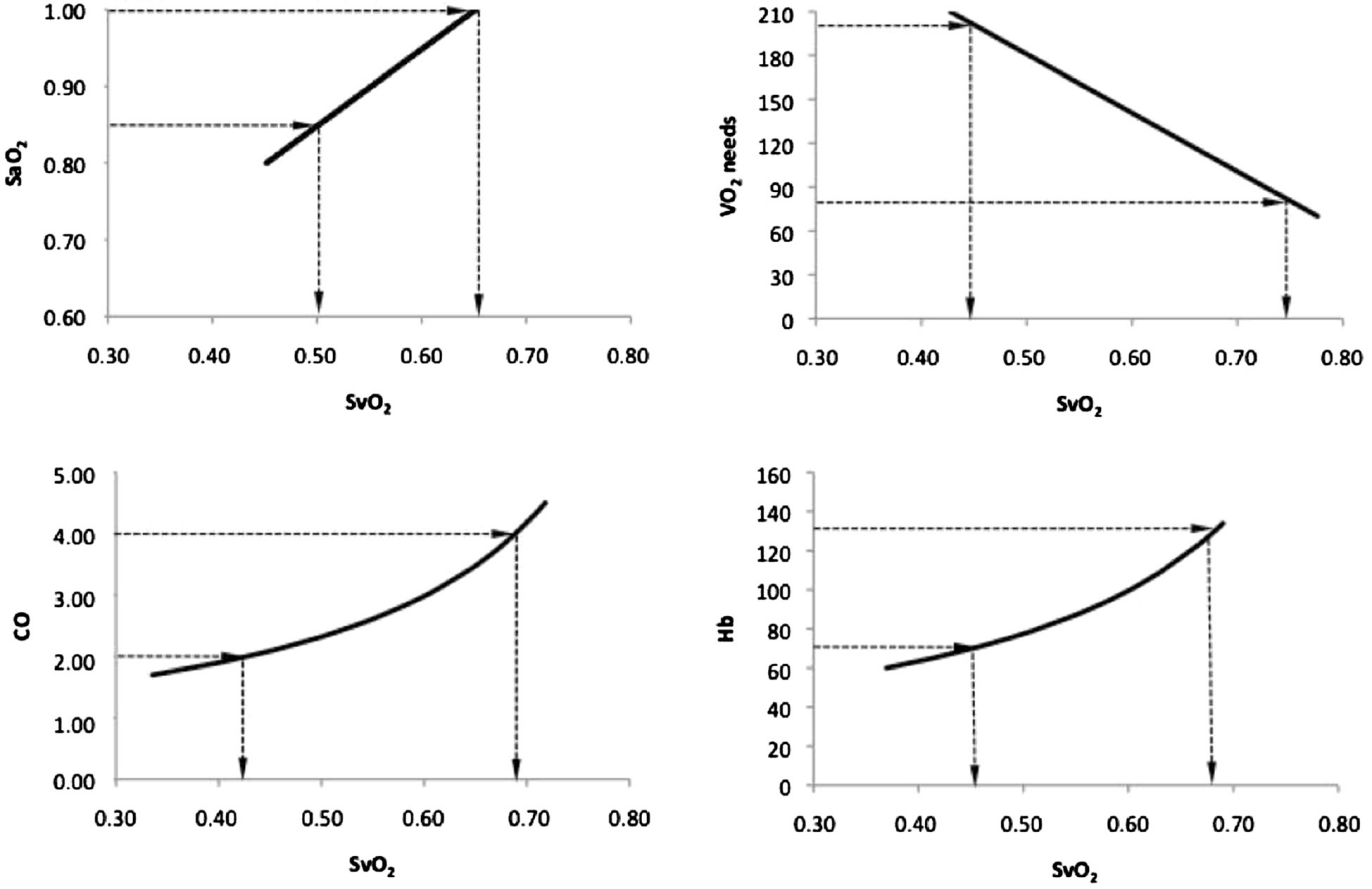
**Relationships between mixed venous oxygen hemoglobin saturation and its components.** To create these curves, we modeled a standard ICU population of 1,000 patients (Excel, Microsoft) with normal distribution of arterial oxygen hemoglobin saturation (SaO_2_; 0.95 ± 0.05 limited to 1) and normal distributions of total body oxygen consumption (VO_2_) needs (140 ± 30 ml/minute.m^2^), cardiac output (CO; 3.0 ± 0.5 L/minute.m^2^) and hemoglobin concentration (Hb; 100 ± 15 g/L). Only one of the four components was sequentially changed (Y-variables) to look at its specific relationship with mixed venous oxygen saturation (SvO_2_) (X-variable). When unchanged, variables were set to their mean value. The horizontal arrows indicate the fluctuations of the Y-variable around its mean value (±2 standard deviations). The vertical arrows show the corresponding fluctuations of SvO_2_. We can see that reference ranges (mean ±2SD) of SaO_2_, VO_2_ needs, CO, and Hb are compensated for by a 26%, 50%, 47%, and 40% change in SvO_2_, respectively. Thus, CO is not necessarily the predominant component of SvO_2_ except when low (left, flat part of the relationship).

For any cell, tissue, and organ, VO_2_ is the difference between arterial and venous O_2_ flows. For the whole body, if we ignore the O_2_ dissolved in the plasma water, which represents only a few percent of the total O_2_ blood content, if we consider that arterial and venous blood flows are represented by the cardiac output (CO), and if we assume that arterial and venous blood flow have a similar hemoglobin concentration (Hb), then we can write:

2 $$ \mathrm{V}{\mathrm{O}}_2=\mathrm{C}\mathrm{O}\times \mathrm{H}\mathrm{b}\times 1.34\times \left(\mathrm{S}\mathrm{a}{\mathrm{O}}_2-\mathrm{Sv}{\mathrm{O}}_2\right) $$

where VO_2_ is in ml/minute.m^2^, CO is in L/minute.m^2^, Hb is in g/L, and arterial oxygen hemoglobin saturation (SaO_2_) and SvO_2_ are the ratios of arterial and venous oxygenated Hb over the total Hb per blood unit and, therefore, dimensionless percentages. The constant 1.34 is the carrying capacity of the oxygenated Hb in milliliters of O_2_ per gram. This equation can be reformulated as a function of each variable, but its reformulation as a function of SvO_2_ is one of the most popular because SvO_2_ measurements are precise, accurate, time responsive and quite easy to monitor [10,11]. Figure 1 shows that the relationships between SvO_2_ and its components are not equivalent and not necessarily linear. As a consequence, a large change in one variable may be compensated by a small change in another - for example, small changes in low CO that are compensated for by large changes in SvO_2_ and large changes in high CO that are compensated for by small changes in SvO_2_:

3 $$ \mathrm{S}\mathrm{v}{\mathrm{O}}_2=\mathrm{S}\mathrm{a}{\mathrm{O}}_2-\mathrm{V}{\mathrm{O}}_2/\left(\mathrm{C}\mathrm{O}\times \mathrm{H}\mathrm{b}\times 1.34\right) $$

If we replace the measured value of VO_2_ by its two hidden components seen in [1], we find:

4 $$ \mathrm{Sv}{\mathrm{O}}_2=\mathrm{S}\mathrm{a}{\mathrm{O}}_2-\mathrm{V}{\mathrm{O}}_2\mathrm{needs}/\left(\mathrm{C}\mathrm{O}\times \mathrm{H}\mathrm{b}\times 1.34\right)+\mathrm{V}{\mathrm{O}}_2\mathrm{gap}/\left(\mathrm{C}\mathrm{O}\times \mathrm{H}\mathrm{b}\times 1.34\right) $$

The purpose of the circulatory system, eventually supported by intensive care, is to nullify the VO_2_ gap; at equilibrium, therefore, Equation 4 can be written:

5 $$ {}^{\mathrm{e}}\mathrm{Sv}{\mathrm{O}}_2=\mathrm{S}\mathrm{a}{\mathrm{O}}_2-\mathrm{V}{\mathrm{O}}_2\kern0.2em \mathrm{needs}/\left(\mathrm{C}\mathrm{O}\times \mathrm{H}\mathrm{b}\times 1.34\right) $$

where ^e^SvO_2_ is the expected SvO_2_ to maintain the equilibrium.

In reality, the ‘two hands of equality’ in Equation 5 fluctuate around the equilibrium. When SvO_2_ is superior to the right hand, a VO_2_ gap is created, and when below, the gap is restored. If we subtract Equation 5 from Equation 4, we can see that the difference SvO_2_ - ^e^SvO_2_ (ΔSvO_2_) is related to the VO_2_ gap:

6 $$ \Delta \mathrm{S}\mathrm{v}{\mathrm{O}}_2=\mathrm{V}{\mathrm{O}}_2\mathrm{gap}/\left(\mathrm{C}\mathrm{O}\times \mathrm{H}\mathrm{b}\times 1.34\right) $$

## Physiological adaptation

Any change in metabolic needs triggers active neuro-hormonal regulation to enable the actual VO_2_ to equalize to the VO_2_ needs as soon as possible. Under basal metabolic conditions, VO_2_ needs depend mostly on age, gender, height and weight [12-14]. In the resting state, physiological changes in the basal metabolism are mostly due to digestion and body temperature. A normal meal usually increases the metabolic rate by 4 to 10% and each degree change in temperature over or under 37°C alters VO_2_ needs by 13% [8]. Consequently, the expected VO_2_ of resting patients may be easily computed or found from normative tables.

**Figure 2:**
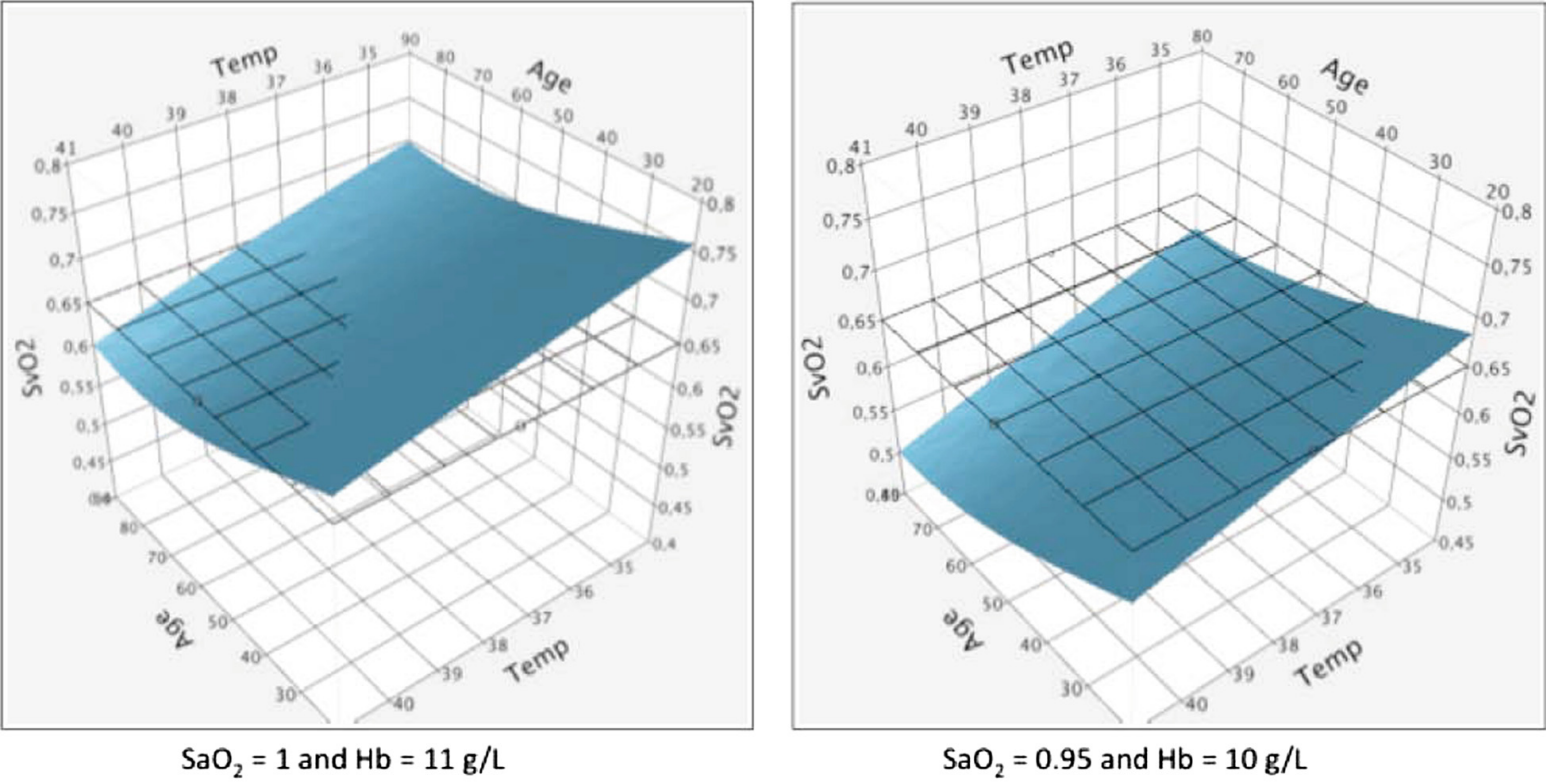
**Mixed venous oxygen hemoglobin saturation adaptation to fluctuations of related variables.** Mixed venous oxygen saturation (SvO_2_) changes were modeled in 1,000 patients using JMP (SAS Institute, Cary, North Carolina, USA). In both panels, we used a random uniform distribution of age (range 20 to 90 years) and temperature (range 34 to 41°C) and the normative tables as modeled by the Hemodyn software® [7,26-29]. This generates a wide range of total body oxygen consumption (VO_2_) needs (70 to 290 ml/minute.m^2^) and their corresponding cardiac output values (2.28 to 4.22 L/minute.m^2^) from which individual values of needed SvO_2_ can be inferred from Equation 2 according to different values of hemoglobin concentration and arterial oxygen hemoglobin saturation. In the left-hand panel, only old and hyperthermic patients have an expected physiological SvO_2_ < 0.65. In the right-hand panel, most patients have an expected SvO_2_ < 0.65 in response to a combined mild decrease in SaO_2_ and hemoglobin concentration. These examples have been created assuming no VO_2_ gap. In case of hypoxia, SvO_2_ values exceed the modeled value, proportionally to the VO_2_ gap following Equation 6.

This VO_2_ adaptation to metabolic needs is primarily achieved by stimulus-induced catecholamine secretion modulating global CO and its distribution. SaO_2_ is maintained close to 1 by the ventilation drive triggered by brain receptors. When low, Hb is also improved, albeit slowly, by iron mobilization, [15] kidney secretion of erythropoietin, [16] and the release of young red blood cells [17,18]. In contrast, no regulatory loop has been observed for maintaining the mixed venous oxygen partial pressure or SvO_2_ within specific ranges. Therefore, SvO_2_ seems to be a variable that passively follows the regulation of its components. For any change in VO_2_ needs, the tissue residual partial pressure of O_2_ represents the final adjustment between O_2_ delivery and uptake and determines the change in SvO_2_. The proportional contributions of CO and SvO_2_ to the changes in VO_2_ strongly depend on body position, blood volume, and the protocol for increasing the requirements. In the following, we will assume that in ICU patients, lying supine, with acceptable blood volume, two-thirds of the change in VO_2_ is achieved by a parallel change in CO and one-third by an anti-parallel change in SvO_2_ [19-23]. This allows us to derive the expected adaptive values of SvO_2_ as a function of age, gender and temperature (Figure 2). The two-thirds to one-third balance, observed in cases of physiologic stress, should be seen as a pivotal value in critical situations. In case of limited CO for any reasons, necessary compensation would be reached by a proportional decrease in SvO_2_ and vice versa.

**Figure 3:**
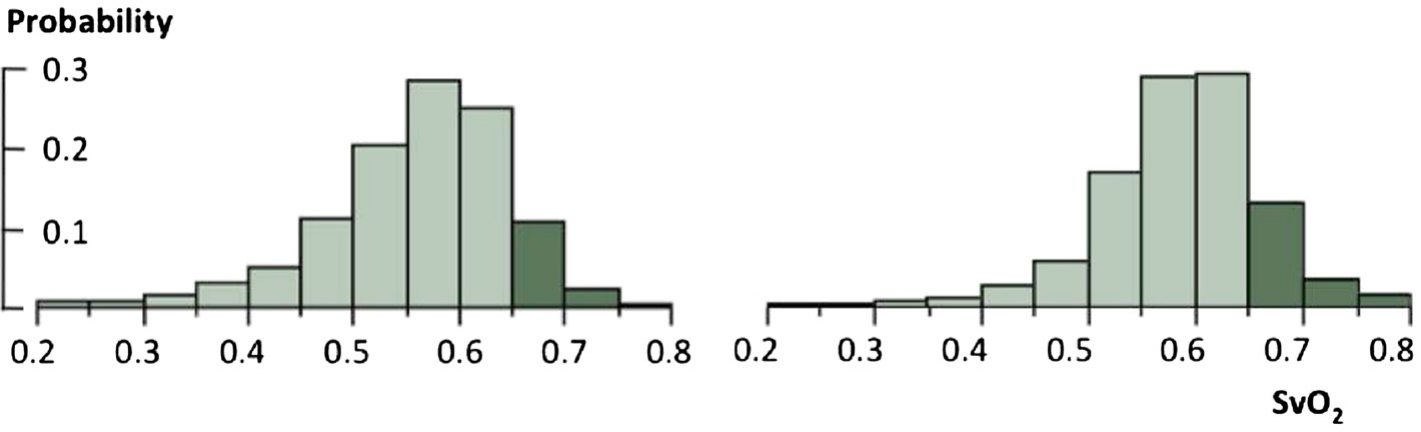
**Frequency distribution of mixed venous oxygen hemoglobin saturation in two populations of patients.** Left: a modeled population of 1,000 patients (JMP, SAS Institute, Cary, North Carolina, USA) as seen in Figure 2 but mimicking a standard ICU population by computing random gender, normal distribution of age (60 ± 12 years), temperature (37.5 ± 1.2°C), hemoglobin concentration (100 ± 15 g/L) and semi-normal distribution of arterial oxygen hemoglobin saturation (1 to 0.05) and assuming no total body oxygen consumption (VO_2_) gap. Right: a real population of post-cardiosurgery unshocked steady state patients (assuming no VO_2_ gap) with a wide range of changes in mixed venous oxygen saturation (SvO_2_) components (15,860 measurements obtained from a previously published study) [64].

The adaptive physiological variability of SvO_2_ that maintains VO_2_ equal to needs can also be shown by its frequency distribution in large populations of steady-state patients (Figure 3). We can see that the expected SvO_2_ is often below 65% in both modeled and real anesthetized patients. In real, non-anesthetized patients, other physiological contributors to metabolic needs, such as digestion, pain, discomfort, stress, inflammation, increased work of breathing, and so on, additionally increase this heterogeneity.

## Pathological changes

When the VO_2_ gap exceeds the tissue O_2_ reserve, the cell moves from aerobic to anaerobic metabolism, leading to tissue hypoxia and dysoxia [24,25]. This can be first observed when the right-hand side of Equation 5 is excessively low (low SaO_2_, high VO_2_ needs, low CO or low Hb), such that SvO_2_ cannot decrease proportionally to maintain the equilibrium and a difference occurs between limited SvO_2_ and very low ^e^SvO_2_.

A second pathological situation is observed in the case of impaired tissue O_2_ convection between hemoglobin and mitochondria, such that the arterial blood flow is not sufficiently unloaded and SvO_2_ increases over ^e^SvO_2_. This can be seen when the O_2_ gradient is low due to a deficit in utilization (for example, from mitochondrial blockage, cyanide poisoning, and so on) and/or when O_2_ tissue diffusion is impaired by an excessive distance or a reduced surface area (for example, in the presence of edema, inflammation, microclots, reduced capillary density, anatomical and/or functional shunts). In septic shock or in late stage shock caused by any mechanism, SvO_2_ cannot decrease sufficiently due to a combination of these elementary mechanisms [7,26-29].

In both situations, the parallel changes in SvO_2_, CO and Hb predicted by Equation 3 may be lost since VO_2_ is expected to increase as well up to its needed value.

Except in experimental conditions, these two pathological situations are usually combined [30]. We can reasonably speculate that, depending on the initial mechanisms of shock, its magnitude, and the adaptive possibilities of each patient, SvO_2_ will cover a wide range of values and will not provide by itself clear indications for guiding therapy. The same considerations will also lead to significant discordance between different studies according to the heterogeneity of their populations for each of the elementary mechanisms described above [31].

## Metrological considerations

The reference method for assessing SvO_2_ requires mixed venous blood sampling through a pulmonary artery catheter and direct measurement of hemoglobin saturation using a multi-wavelength spectrophotometer (co-oximeter) [32]. When the blood sampling procedure is correct and the sample is immediately analyzed using a properly calibrated co-oximeter, the SvO_2_ measurement is accurate (bias <0.5%), precise (2 standard deviations (SD)/mean =1.3%) and linear (R^2^ = 1) [33,34]. Even with such good performance indices, however, the least significant change in a unique measurement (2√2 × 2SD/mean) is 3.7%, meaning that a SvO_2_ value of 65% needs to change to >68.7% or <61.3% to have a 95% chance of being real.

Continuous monitoring of SvO_2_ using a fiber-optic sensor placed at the tip of a pulmonary catheter has acceptable accuracy (bias <1%) when properly calibrated and recalibrated using a co-oximeter [35]. The precision is necessarily lower (2SD/mean >5%) [10,11], but is compensated by a very fast response (almost instantaneous) [36], allowing averaging of several elementary measurements (N) in a few milliseconds and decreasing the standard error of the mean (2SD/√N). Averaging 10 elementary measurements when continuously monitoring SvO_2_ allows the same least significant changes to be achieved as when analyzing a unique mixed venous blood sample.

At the turn of the century, it was suggested that ScvO_2_ should be used as a surrogate of SvO_2_, owing to the fact that it is easier and less invasive to insert a central line than a pulmonary catheter. ScvO_2_ is similar to SvO_2_ in normal patients, being about 2 to 3% lower because many of the vascular circuits that drain into the inferior vena cava may have non-oxidative phosphorylation (renal, portal, hepatic blood flows) and therefore extract less O_2_ [37,38]. However, several studies have shown that ScvO_2_ may not predict SvO_2_ in patients suffering shock, depending on the O_2_ flows and O_2_ extractions of the different tissue compartments, and where measurements are taken [39-43]. The coefficient of variation (2SD/mean) between ScvO_2_ and SvO_2_ may exceed ±20% [44,45]. Even paired changes in ScvO_2_ and SvO_2_ are not necessarily parallel; they were only found in 55% of cases in one study [45]. It is only when considering trend lines that changes in ScvO_2_ and SvO_2_ become more consistent [46].

## Clinical evidence

Finally, there is a huge body of evidence (often with a degree of mathematical evidence) that SvO_2_ values vary widely in ICU patients, either for physiological, pathological or metrological reasons. Therefore, the appropriate SvO_2_ for achieving an adequate body energy supply is specific to each individual patient and to its specific time-evolving situation. There is no basic evidence for targeting any clear-cut SvO_2_ value. From the considerations listed above, it seems more appropriate to tune a multivariate compromise represented by an acceptable range of the four SvO_2_ component trend lines with the objective of fulfilling global and local metabolic needs. This compromise must be assessed in terms of predicted benefit and risk of any change.

A variety of clinical evidence has provided us with a message in accordance with these fundamentals, showing no interest in targeting specific values of SvO_2_ and/or ScvO_2_ in large populations of patients [47-49]. However, other studies have presented indisputable evidence that targeting a specific value of SvO_2_/ScvO_2_ can be of interest for lactate clearance [50], morbidity [51], and mortality [52]. Accordingly, the Surviving Sepsis Campaign recommends maximizing mixed or central venous oxygen saturation [2]. The contradiction is apparent. As previously mentioned and detailed above, all results can be predicted by the homogeneity/heterogeneity of the patient population.

**Figure 4:**
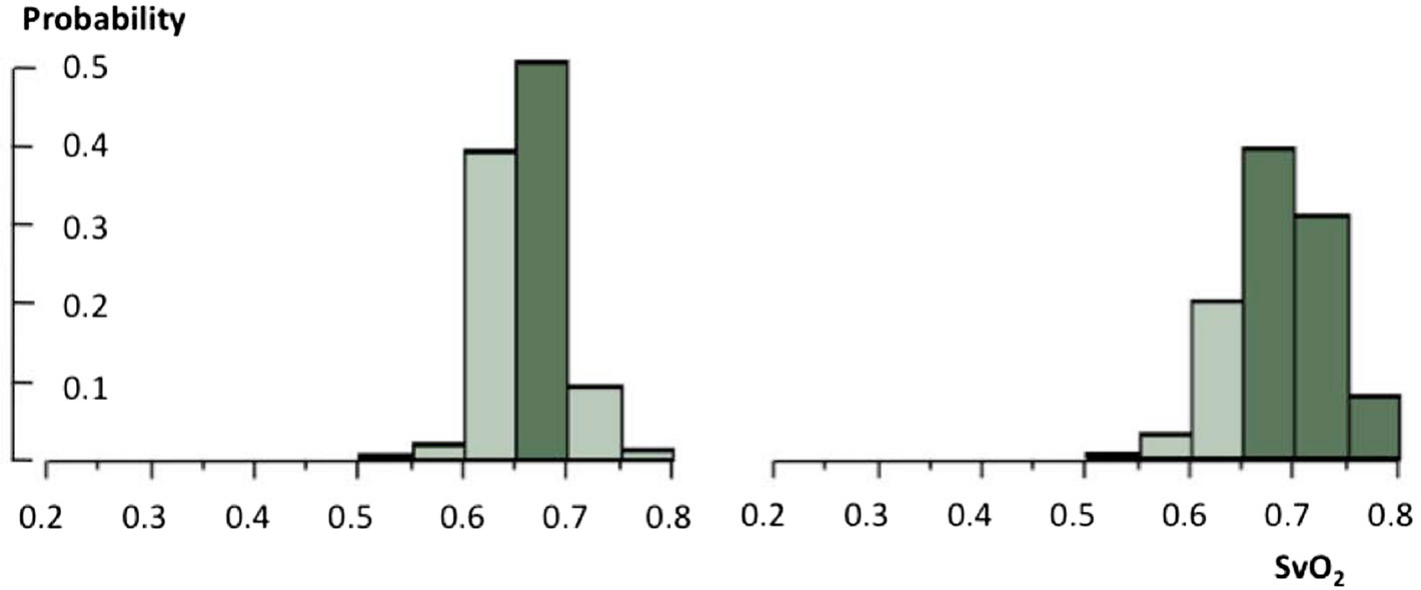
**Frequency distribution of mixed venous oxygen hemoglobin saturation in two populations of ‘optimized’ patients according to the protocol of Rivers and colleagues** **[** 52 **].** The left-hand panel represents the population of 1,000 patients as seen in Figure 3 but total body oxygen consumption needs and cardiac output (CO) were set at 120% and 130% of the expected values at basal metabolism for modeling septic conditions. Other variables were distributed as reported by Rivers and colleagues: age =67 ± 17.4 years, 50.8% male sex ratio, temperature =35.9 ± 3.2°C, hemoglobin concentration (Hb) >100 ± 15 g/L and arterial oxygen hemoglobin saturation (SaO_2_) >0.93. The right-hand panel represents the same real population of post-cardiosurgery patients as shown in Figure 3 but restricted to CO >1.3 basal values, Hb >100 ± 15 g/L and SaO_2_ > 0.93 (8,067 measurements). SvO_2_, mixed venous oxygen saturation.

In the Rivers and colleagues’ study [52], which is the main reference for the hemodynamic recommendations of the Surviving Sepsis Campaign, the population was homogenized as much as possible before targeting the ScvO_2_. The study included early septic shock before ICU admission, and thus compared patients at the same phase of pathological evolution. In addition, the therapeutic protocol, including sedation, mechanical ventilation and fraction of inspired oxygen adjustment, resulted in a decrease in VO_2_ needs and the maximization of SaO_2_. Hemoglobin was also increased when necessary by blood transfusion. Heart rate and central venous pressure were optimized, thus improving CO as much as possible. Under these conditions, if a low ScvO_2_ was found instead of an expected high value, even taking into consideration a possible discordance between ScvO_2_ and SvO_2_, the probability of insufficient CO was high and use of inotropes was consistent with the basic physiology. This is illustrated in Figure 4, where we used the same populations as shown in Figure 3 but with the distributions of the SvO_2_ components mimicking those of the study by Rivers and colleagues. In these homogenized populations, the SvO_2_ distribution is obviously narrower. If Hb, SaO_2_ and VO_2_ are homogenized, ScvO_2_ and/or SvO_2_ are necessarily more influenced by CO.

From the modeled population seen in Figure 4, we can simulate the increase in CO required to reach a target SvO_2_ of >65% (presumably equivalent to ScvO_2_ > 70%). Such an increase would be required in 39.5% of the patients, up to 1.2 L/minute.m^−2^ (average 0.17 ± 0.16), which seems to be a reasonable objective.

However, the Surviving Sepsis Campaign recommended a target Hb of 70 to 90 g/L whereas in Rivers and colleagues’ study the hematocrit was increased up to 30%, which represents an Hb >100 g/L. This has major consequences for SvO_2_. If we consider again the population modeled in Figure 4, reducing Hb from 100 to 90 g/L displaces the frequency distribution leftwards and it would be more difficult to target SvO_2_ to >65%. The simulation shows that an increase in CO would be necessary for 79.5% of the patients, up to 1.7 L/minute.m^2^, with an average increase of 0.39 ± 0.24. If targeting Hb to 70 g/L, an increase in CO would be necessary for 98.7% of the patients, up to 2.7 L/minute.m^2^ with a mean increase of 1.30 ± 0.44. All these estimations are derived with constant VO_2_ needs, ignoring the caloric effects of increasing CO [53]. Therefore, targeting the same SvO_2_ objective as Rivers and colleagues without targeting the same Hb has strong consequences for CO stimulation. Finally, SvO_2_ must be viewed as a compromise. Increasing Hb may have favorable [54] or detrimental effects [55]. Increasing CO may also have positive [56,57] or negative results [58-61]. The final decision depends, therefore, on the specific conditions and limitations of each patient. This statement is reinforced by two recent reports. In the study of Jones and colleagues [62] management to increase lactate clearance was equivalent to targeting specific ScvO_2_ values in septic shock. Moreover, the ProCESS trial has shown that, in academic hospitals, the Rivers and colleagues’ protocol was not superior to usual care despite significant increases in blood transfusion, dobutamine and vasopressor use [63]. The comparable mortality may only be explained by an absence of impact on mortality of these interventions, which seems unlikely, or by the fact that targeting a unique ScvO_2_ value in heterogeneous patients may balance positive and negative effects. Whether a unique SvO_2_ or ScvO_2_ goal would be beneficial or not depends, therefore, on the quality of care in the control group and on the inter-individual dispersion of the difference between the target and the optimal ScvO_2_ value allowing VO_2_ needs to be met. We have seen that this optimal value may be far from a fixed target. These results should not discourage us from monitoring SvO_2_ or ScvO_2_ but encourage us to include these variables in a multimodal analysis.

## Conclusion

Basic physiology tells us that SvO_2_ is not a regulated variable but an adaptive variable depending on four elementary regulated components: VO_2_ needs, SaO_2_, Hb and CO. Consequently, SvO_2_ is widely fluctuating. There is no physiological argument for targeting particular values of SvO_2_ (or its surrogate ScvO_2_) by specific interventions except in homogenized populations, where optimizing one to three of the four SvO_2_ components may yield a clear dependency with the fourth one. This explains the apparently contradictory results observed in large studies where CO was increased up to specific SvO_2_ thresholds and confirms the basic physiology predicting large inter-patient variability. Although a systematic SvO_2_ goal-oriented protocol can be statistically profitable before ICU admission, one would expect from any trained intensivist a more sophisticated, multivariate approach and a determination of the best compromise between SvO_2_ and its components_,_ taking into account the specific constraints of each individual patient.

## Abbreviations

CO: Cardiac output
^e^SvO_2_: Expected SvO_2_
Hb: Hemoglobin concentration
SaO_2_: Arterial oxygen hemoglobin saturation
ScvO_2_: Central venous oxygen saturation
SD: Standard deviation
SvO_2_: Mixed venous oxygen saturation
VO_2_: Total body oxygen consumption
